# From participation to systematization: A scoping review of theoretical frameworks guiding process evaluations in community mental health interventions

**DOI:** 10.1371/journal.pone.0354732

**Published:** 2026-07-28

**Authors:** Rainer Mere, Merike Sisask, Peeter Värnik, Chantal Van Audenhove

**Affiliations:** 1 School of Governance, Law and Society (SOGOLAS), Tallinn University, Tallinn, Estonia; 2 Estonian-Swedish Mental Health and Suicidology Institute (ERSI), Tallinn, Estonia; 3 LUCAS–Centre for Care Research and Consultancy, KU Leuven, Leuven, Belgium; Universiti Pertahanan Nasional Malaysia, MALAYSIA

## Abstract

**Background:**

Process evaluations are essential for understanding how community mental health interventions are implemented and why they succeed or fail. While numerous theoretical frameworks exist to guide such evaluations, the landscape of framework use in mental health contexts remains uncharted, limiting methodological guidance for researchers and practitioners.

**Methods:**

Scoping review following Preferred Reporting Items for Systematic Reviews and Meta-Analyses extension for Scoping Reviews guidelines. Systematic searches of three databases (PubMed, Web of Science, EBSCOhost) identified 1,143 records, from which process evaluations of community mental health interventions published between 2006 and 2025 were selected. Data extraction captured framework characteristics, study context, and methodological features. Frameworks were inductively categorized into a hierarchical typology based on theoretical orientation and stated function.

**Results:**

Eighty-three studies met inclusion criteria, employing 54 distinct primary frameworks organized into 14 categories. Implementation science frameworks dominated (39.8%), followed by process evaluation-specific frameworks (8.4%), behavior change theories (8.4%), and participatory approaches (6.0%). A marked temporal shift was observed: participatory and community-based frameworks characterized early publications (23.8% in 2006–2015) but were absent as primary frameworks after 2015, while implementation science frameworks rose from 14.3% to over 50% of studies. Geographic concentration in North America (54.2%) and high-income countries (83.1%) was pronounced.

**Conclusions:**

The field has undergone a paradigm shift from community-engaged to systematized evaluation approaches. While framework consolidation offers methodological consistency, the decline of participatory approaches raises questions about the role of community voice in evaluation design. We propose a typology to guide framework selection and identify priorities for methodological development in community mental health process evaluation.

## Introduction

Mental health interventions with demonstrated efficacy under controlled conditions frequently fail to achieve comparable outcomes when implemented in community settings. This gap between efficacy and effectiveness represents one of the most persistent challenges in mental health services research, with estimates suggesting that it takes an average of 17 years for research evidence to reach routine practice [1,2], and even then, implementation with fidelity remains a persistent challenge [3]. Process evaluation (the systematic examination of how interventions are delivered, received, and experienced) offers a critical lens for understanding this implementation gap, revealing the mechanisms through which interventions succeed or fail in real-world contexts.

The value of process evaluation in mental health has gained increasing recognition over the past two decades. The Medical Research Council’s (MRC) influential guidance on evaluating complex interventions, first published in 2000 and updated in 2008 and 2021 [4–6], established process evaluation as an essential complement to outcome evaluation, arguing that understanding implementation is necessary for interpreting trial results and supporting intervention scale-up. Dedicated guidance on process evaluation methodology followed in 2015 [7]. This recognition has catalyzed a substantial body of methodological development, with researchers proposing numerous theoretical frameworks to guide the design and conduct of process evaluations [8].

Theoretical frameworks serve multiple functions in process evaluation. They direct attention to specific aspects of implementation, provide conceptual categories for organizing data, enable comparison across studies, and support the accumulation of knowledge about implementation processes. The choice of framework shapes what questions are asked, what data are collected, and ultimately what conclusions can be drawn about intervention implementation. Given this influence, understanding which frameworks researchers employ and why has significant implications for the field’s development.

Yet despite the proliferation of frameworks for guiding process evaluation, researchers face a fragmented landscape with limited guidance for framework selection. Previous reviews have catalogued frameworks available in implementation science broadly [8,9], but none have systematically examined which frameworks are actually being used in process evaluations of mental health interventions specifically. This gap is consequential because community mental health interventions present distinct implementation challenges. They involve complex behavior change across multiple levels including individual, community, and system and operate in complex social environments where contextual factors exert substantial influence on implementation processes and outcomes [10]. They require attention to stigma and cultural context, often depend on relationships and trust that develop over time, and frequently aim to reach populations who do not access traditional mental health services. The community itself may be both the setting for intervention delivery and a target of change, as in community-wide suicide prevention initiatives or depression awareness campaigns. These characteristics suggest that process evaluation in community mental health may require frameworks attuned to community dynamics, participation, and context in ways that generic implementation frameworks may not address. Whether the frameworks developed primarily in other health domains adequately address these distinctive features remains an open question.

The present scoping review aims to address this gap by mapping the theoretical landscape of frameworks used to guide process evaluations in community mental health interventions. Our specific objectives were:

1. To identify and describe the theoretical frameworks used to guide process evaluations of community mental health interventions published between 2006 and 2025
2. To develop a typology for categorizing these frameworks based on their theoretical orientation and stated function
3. To examine temporal trends and geographic patterns in framework adoption
4. To identify implications for framework selection and methodological development in the field

## Methods

### Design

We conducted a scoping review following the methodological framework of Arksey and O’Malley [11] as enhanced by Levac and colleagues [12], and reported according to the Preferred Reporting Items for Systematic Reviews and Meta-Analyses extension for Scoping Reviews (PRISMA-ScR) [13]. Scoping review methodology was selected over systematic review because our aim was exploratory: to map the breadth of framework use across a heterogeneous literature rather than to synthesize findings around a narrowly defined question. This approach is appropriate when examining how research is conducted within a field and when the goal is to identify key concepts and gaps in the literature. This review was not prospectively registered; however, the research questions and analytical approach were established prior to data extraction.

### Eligibility criteria

Studies were eligible for inclusion if they: (1) reported a process evaluation or implementation evaluation of a mental health intervention; (2) described an intervention delivered in a community-based or community-accessible setting, defined as settings outside of inpatient psychiatric facilities and primary care clinical encounters (primary care was excluded to maintain focus on non-clinical community settings where implementation challenges differ qualitatively from healthcare delivery contexts); (3) used, referenced, or explicitly discussed the absence of a theoretical or conceptual framework to guide evaluation design or analysis; (4) were published in English between January 2006 and April 2025; and (5) appeared in peer-reviewed journals. The 2006 start date was selected to capture the period following the initial MRC guidance on evaluating complex interventions, which marked a significant inflection point in attention to process evaluation methodology. Community-based settings were broadly defined to include schools, workplaces, community organizations, religious institutions, and other non-clinical settings where mental health interventions are delivered.

We included studies addressing three overlapping domains: broader mental health interventions (targeting general mental wellbeing, resilience, common mental disorders, or multiple conditions), depression-focused interventions, and suicide prevention interventions. No age restrictions were applied, so eligible interventions spanned all age groups, from adolescents in school-based settings to adults in workplace and community settings. Studies were excluded if they: (1) evaluated interventions delivered exclusively in inpatient or residential treatment settings; (2) focused solely on clinical treatment without community components; (3) were framework development papers without empirical application; (4) were conference abstracts, dissertations, or grey literature; or (5) reported only outcome evaluation without process evaluation components.

### Information sources and search strategy

We searched PubMed, Web of Science Core Collection, and EBSCOhost (including APA PsycArticles and Academic Search Ultimate) from January 2006 to April 2025. The search strategy combined terms for process evaluation and implementation (“process evaluation,” “implementation framework,” “theoretical framework,” “program evaluation,” “implementation science”), community settings (“community mental health,” “community-based,” “population-based,” “public mental health”), and mental health conditions (“mental health,” “depression,” “suicide prevention,” “suicidal behavior”). The complete search strings for each database are provided in S1 File.

### Selection process

Following import to Rayyan systematic review software, duplicate records were removed automatically. Titles and abstracts were screened by the first author against inclusion criteria, with conservative inclusion of borderline cases. To enhance screening reliability, the second author independently reviewed a subset of retained records, assessing relevance to community-based mental health interventions with explicit process evaluation components; records with ambiguous community or process evaluation focus were discussed and resolved through consensus. Full texts of remaining articles were assessed for final eligibility by the first author, with uncertain cases resolved through discussion between the first two authors.

### Data extraction

A standardized data extraction form was developed in Microsoft Excel and pilot-tested on a subset of ten studies. The form captured: study identification (authors, year, title); geographic region and country income level (using World Bank 2024 classifications); intervention domain (broader mental health, depression-focused, or suicide prevention); study design (mixed methods, qualitative, case study, protocol, review, or theoretical paper); whether the process evaluation was embedded within a trial and trial type; primary theoretical framework used; secondary frameworks mentioned; number of frameworks referenced; and coder notes documenting extraction decisions. Data extraction was conducted by the first author. Extraction decisions, particularly regarding framework categorization and identification of primary versus secondary frameworks, were reviewed and discussed with the second author at regular intervals throughout the extraction process to ensure consistency.

### Framework typology development

The development of our framework typology followed an iterative, inductive process. Initial extraction captured the specific framework names as reported by study authors. Through iterative review, we grouped frameworks into categories based on their theoretical roots, stated purposes, and level of analysis. Initial categorization yielded fewer categories, which were progressively refined and expanded to 14 as distinct theoretical traditions and functional orientations were identified within broader groupings. For example, implementation science frameworks were subdivided into determinant, evaluation, and process subtypes based on their distinct functions as articulated in Nilsen’s [8] taxonomy. We further organized the 14 categories into broader typological groupings to facilitate synthesis. Category definitions were refined through discussion between the first two authors until consensus was reached. Categorization decisions were documented in a coding manual to ensure transparency and support reproducibility.

Where studies referenced multiple frameworks, we identified a primary framework using a hierarchical decision rule: (1) the framework explicitly designated as ‘primary’ or ‘guiding’ by the authors; if not stated, (2) the framework used to structure the methods section or data collection instruments; if still unclear, (3) the framework used to organize the presentation of findings. In rare cases where these criteria did not resolve ambiguity, categorization was determined through discussion between the first two authors. Secondary frameworks were recorded separately.

### Data synthesis and analysis

Descriptive statistics characterized the distribution of study features and framework use. For temporal analysis, we divided the study period into three phases: an early period (2006–2015), the decade following initial MRC guidance; a middle period (2016–2020), during which implementation science underwent significant growth and institutionalization [14,15]; and a recent period (2021–2025) representing contemporary practice. The 2015 midpoint corresponds with publication of dedicated MRC process evaluation guidance [7], allowing examination of framework use before and after this influential methodological publication.

## Results

### Study selection

**Fig 1:**
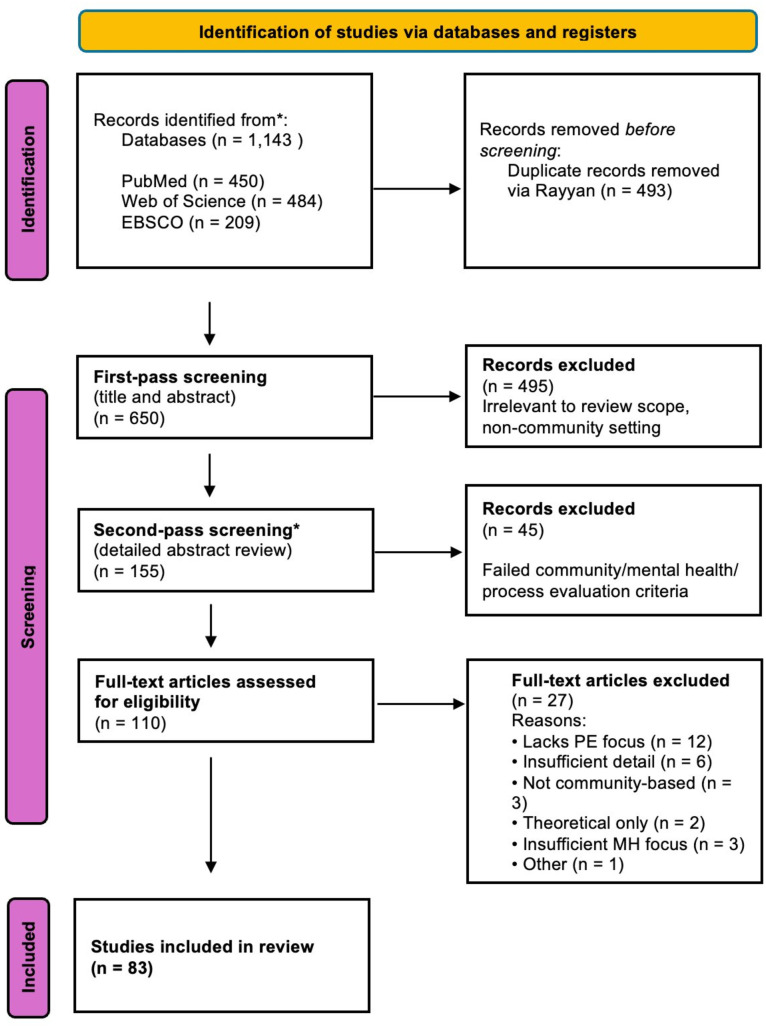
PRISMA-ScR flow diagram illustrating the study selection process for the scoping review.

Database searches identified 1,143 records across three databases. After duplicate removal and two-phase screening, 83 studies met all inclusion criteria (Fig 1).

### Study characteristics

Table 1 presents the characteristics of included studies. Publication volume increased substantially over time, with nearly half of all studies (44.6%) published in 2021–2025, reflecting growing attention to process evaluation in community mental health.

**Table 1:** Characteristics of included studies (N = 83).

Characteristic	n	%
Geographic Region		
North America	45	54.2
Europe	19	22.9
Asia-Pacific	8	9.6
Middle East/Africa	5	6.0
Latin America	3	3.6
Multi-region	3	3.6
Country Income Level		
High-income	69	83.1
Upper-middle income	7	8.4
Low/lower-middle income	5	6.0
Mixed	2	2.4
Intervention Domain		
Broader mental health	57	68.7
Depression-focused	13	15.7
Suicide prevention	13	15.7
Study Design		
Mixed methods	38	45.8
Qualitative	21	25.3
Case study	14	16.9
Protocol	6	7.2
Review/Theoretical	4	4.8
Trial-Embedded Process Evaluation		
Standalone (not trial-embedded)	65	78.3
Embedded in trial	18	21.7
Publication Period		
2006–2015	21	25.3
2016–2020	25	30.1
2021–2025	37	44.6

Geographic distribution was heavily concentrated in high-income, Western settings: North America and Europe together accounted for over three-quarters of studies (77.1%), while low- and middle-income countries (LMIC) contributed only 14.4%. This pattern has implications for the generalizability of framework-based knowledge that we address in the Discussion.

The majority of studies addressed broader mental health interventions (68.7%) rather than condition-specific programs, and most were standalone investigations (78.3%) rather than trial-embedded evaluations. Mixed methods designs predominated (45.8%), consistent with the multidimensional nature of process evaluation.

### Framework typology

The 83 included studies employed 54 distinct primary frameworks, which we organized into 14 categories based on theoretical orientation and stated purpose (Table 2). The typology distinguishes between frameworks designed specifically for implementation research, frameworks drawn from other theoretical traditions, and hybrid approaches combining multiple perspectives.

**Table 2:** Typology of frameworks used in process evaluations of community mental health interventions (N = 83).

#	Framework Category	n	%	Exemplar Frameworks	Core Function
1	Determinant Frameworks (IS)	14	16.9	CFIR, TDF, EPIS, iPARIHS	Identify factors affecting implementation
2	Evaluation Frameworks (IS)	10	12.0	RE-AIM, PRISM, Proctor outcomes	Structure outcome assessment
3	Process Frameworks (IS)	9	10.8	NIRN/AIF, Fixsen stages	Guide implementation stages
4	Process Evaluation Specific	7	8.4	MRC Framework, Linnan & Steckler	Comprehensive PE guidance
5	Behavior Change Theories	7	8.4	TPB, COM-B/TDF, TTM	Explain individual-level change
6	Other Frameworks	8	9.6	Intervention-specific, organizational	Various
7	Hybrid/Multiple Frameworks	5	6.0	Combined approaches	Integrate perspectives
8	Participatory Approaches	5	6.0	CBPR, CPPR, KMb	Center community voice
9	Program Theory	4	4.8	Theory of Change, Realist Evaluation	Articulate causal logic
10	No Explicit Framework	4	4.8	N/A	Inductive analysis only
11	Public Health/Prevention Model	3	3.6	Universal-selective-indicated	Population-level framing
12	Study-Developed Frameworks	3	3.6	BC4, Empowerment implementation	Novel frameworks
13	Quality/Fidelity Frameworks	3	3.6	Donabedian, 5-component model	Assess implementation quality
14	Health Services/Utilization	1	1.2	Andersen’s Behavioral Model	Service access focus

*Note. IS = Implementation Science; CFIR = Consolidated Framework for Implementation Research; TDF = Theoretical Domains Framework; EPIS = Exploration, Preparation, Implementation, Sustainment; iPARIHS = integrated Promoting Action on Research Implementation in Health Services; RE-AIM = Reach, Effectiveness, Adoption, Implementation, Maintenance; PRISM = Practical, Robust Implementation and Sustainability Model; NIRN/AIF = National Implementation Research Network/Active Implementation Frameworks; MRC = Medical Research Council; TPB = Theory of Planned Behavior; COM-B = Capability, Opportunity, Motivation–Behavior; TTM = Transtheoretical Model; CBPR = Community-Based Participatory Research; CPPR = Community-Partnered Participatory Research; KMb = Knowledge Mobilization. One meta-level review study examining framework use was categorized under ‘Other Frameworks.’*

Implementation science frameworks were the most prevalent category overall (39.8%, n = 33). Within this category, determinant frameworks such as the Consolidated Framework for Implementation Research (CFIR) were most common (16.9%), followed by evaluation frameworks like the Reach, Effectiveness, Adoption, Implementation, Maintenance (RE-AIM) framework (12.0%) and process frameworks including the Active Implementation Frameworks (10.8%).

Process evaluation-specific frameworks, particularly the MRC Framework, were used in 8.4% of studies. Behavior change theories appeared with equal frequency (8.4%), typically applied to understand participant-level responses to interventions.

Notably, five studies (6.0%) employed participatory approaches, specifically Community-Based Participatory Research (CBPR), Community-Partnered Participatory Research (CPPR), or Knowledge Mobilization frameworks, as their primary evaluation framework. These frameworks position community members as co-evaluators rather than solely as evaluation subjects, representing a fundamentally different orientation to the evaluation enterprise. As detailed below, this category showed the most dramatic temporal pattern of any in our typology.

Four studies (4.8%) explicitly reported using no theoretical framework, instead employing purely inductive qualitative analysis. The remaining studies distributed across program theory approaches (4.8%), public health models (3.6%), study-developed frameworks (3.6%), quality/fidelity frameworks (3.6%), and other categories.

Seventy-three percent of studies (n = 61) referenced at least one secondary framework in addition to their primary framework, suggesting that researchers frequently draw on multiple theoretical perspectives even when one framework serves as the primary organizing structure.

### Individual framework frequency

Despite the dominance of implementation science as a category, individual framework use remained highly fragmented (Table 3). Only three frameworks appeared in more than five studies: RE-AIM [16] (n = 9), CFIR [17] (n = 8), and the MRC Framework [7] (n = 6). Together, these accounted for only 27.7% of studies. The remaining 60 studies distributed across 51 different frameworks, with 45 frameworks appearing in only a single study. This fragmentation persists despite categorical consolidation around implementation science.

**Table 3:** Most frequently used primary frameworks (n ≥ 3).

Framework	n	Framework Category
RE-AIM	9	IS – Evaluation
CFIR	8	IS – Determinants
MRC Framework	6	Process evaluation specific
Theory of Planned Behavior	4	Behavior change theory
Framework for Dissemination	3	IS – Process

*Note. IS = Implementation Science*

### Temporal trends in framework use

**Fig 2:**
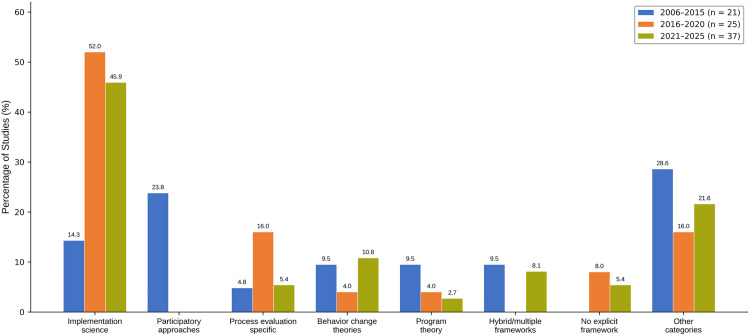
Temporal trends in framework category use across three time periods (2006–2015, 2016–2020, 2021–2025). *Values represent the percentage of studies within each time period using each framework category as their primary framework. Smaller categories are combined under ‘Other categories’ (see* Table 4 *note for composition).*

Fig 2 and Table 4 present the distribution of framework categories across three time periods. Two patterns stand out.

**Table 4:** Temporal trends in framework category use.

Framework Category	2006–2015 (n = 21)	2016–2020 (n = 25)	2021–2025 (n = 37)
IS – Determinant frameworks	1 (4.8)	5 (20.0)	8 (21.6)
IS – Evaluation frameworks	0 (0.0)	3 (12.0)	7 (18.9)
IS – Process frameworks	2 (9.5)	5 (20.0)	2 (5.4)
Implementation science total	3 (14.3)	13 (52.0)	17 (45.9)
Process evaluation specific	1 (4.8)	4 (16.0)	2 (5.4)
Behavior change theories	2 (9.5)	1 (4.0)	4 (10.8)
Participatory approaches	5 (23.8)	0 (0.0)	0 (0.0)
Hybrid/Multiple frameworks	2 (9.5)	0 (0.0)	3 (8.1)
Program theory	2 (9.5)	1 (4.0)	1 (2.7)
No explicit framework	0 (0.0)	2 (8.0)	2 (5.4)
Other categories	6 (28.6)	4 (16.0)	8 (21.6)

*Note. Values represent n (% of studies within time period). Framework categories follow the typology presented in* Table 2*. Other categories include: Other frameworks (n = 8), Public health/Prevention model (n = 3), Study-developed frameworks (n = 3), Quality/Fidelity frameworks (n = 3), and Health services/Utilization (n = 1).*

First, participatory approaches characterized nearly one-quarter of early publications (23.8% in 2006–2015) but were no longer used as primary organizing frameworks after 2015. All five studies using participatory frameworks as their primary approach were published between 2006 and 2015. Notably, however, CBPR and related frameworks continued to appear as secondary frameworks in eight later studies, suggesting a repositioning from organizing framework to supplementary consideration rather than wholesale abandonment. This shift in the structural role of participatory approaches is the most striking temporal finding in our data.

Second, implementation science frameworks showed dramatic growth, rising from 14.3% of studies in 2006–2015 to 52.0% in 2016–2020, and maintaining their dominant position at 45.9% in 2021–2025. Within implementation science, determinant frameworks grew steadily across all periods, evaluation frameworks emerged primarily in later periods, and process frameworks peaked in the middle period before declining.

Framework consolidation was assessed by calculating the ratio of unique frameworks to total studies in each period. This ratio fell from 1.00 in 2006–2015 (every study used a different framework) to 0.60 in 2016–2020, indicating substantial consolidation. The ratio rose slightly to 0.68 in 2021–2025, suggesting modest diversification while remaining far more consolidated than the early period.

### Geographic and economic patterns

Table 5 presents framework category use by geographic region. Three patterns warrant note.

**Table 5:** Framework category use by geographic region.

Framework Category	North America (n = 45) n (%)	Europe (n = 19) n (%)	Asia-Pacific (n = 8) n (%)	Other (n = 11) n (%)
Implementation science frameworks	19 (42.2)	4 (21.1)	4 (50.0)	6 (54.5)
Process evaluation specific	0 (0.0)	4 (21.1)	1 (12.5)	2 (18.2)
Participatory approaches	5 (11.1)	0 (0.0)	0 (0.0)	0 (0.0)
Behavior change theories	4 (8.9)	3 (15.8)	0 (0.0)	0 (0.0)
Other categories	17 (37.8)	8 (42.1)	3 (37.5)	3 (27.3)

*Note. Other includes Middle East/Africa (n = 5), Latin America (n = 3), and multi-region studies (n = 3).*

First, all five studies using participatory approaches originated from North America, suggesting this tradition may be particularly rooted in American community psychology and public health contexts.

Second, the MRC Framework and other process evaluation-specific frameworks were used predominantly in European studies (4 of 7 studies, 57%), consistent with the British origins of MRC guidance.

Third, implementation science frameworks were prevalent across all regions but showed particular dominance outside Europe, accounting for over 50% of studies in Asia-Pacific and other regions. This pattern may reflect the international reach of implementation science training and infrastructure relative to more locally-rooted traditions.

### Framework use by domain

Framework category distribution varied modestly across intervention domains. Depression-focused studies (n = 13) showed stronger representation of implementation science frameworks (46.2%) and accounted for 2 of the 5 studies using participatory approaches as their primary framework, with the remaining 3 being broader mental health studies. Suicide prevention studies (n = 13) were notably diverse in framework use, spanning MRC Framework, implementation science, and public health models. Broader mental health studies largely followed the overall distribution pattern.

## Discussion

### Summary of principal findings

This scoping review mapped the theoretical landscape of frameworks used in process evaluations of community mental health interventions over nearly two decades. We identified 83 studies employing 54 distinct primary frameworks, which we organized into a typology of 14 categories. Implementation science frameworks dominated the field (39.8%), with determinant frameworks like CFIR and evaluation frameworks like RE-AIM emerging as the most commonly used individual approaches. However, despite categorical consolidation, substantial fragmentation persists at the individual framework level, with most frameworks appearing in only one or two studies.

The most striking finding was the marked temporal shift in framework orientation. Participatory approaches, which centered community voice in evaluation design, characterized nearly one-quarter of early publications but were no longer used as primary organizing frameworks after 2015, though they continued to appear as secondary frameworks within implementation science-driven studies. Simultaneously, implementation science frameworks rose from 14% to over 50% of studies. This shift from “participation to systematization” represents a fundamental reorientation in how researchers approach process evaluation in community mental health.

### A paradigm shift: From participation to systematization

We use the term ‘paradigm shift’ in a specific, bounded sense, not to invoke a Kuhnian replacement of one unified theory by another, but to describe a fundamental reconfiguration in how process evaluation is conceptualized and conducted in community mental health research. The shift we identify concerns which questions are prioritized, which theoretical tools are treated as standard, and where epistemic authority is located in the evaluation process.

This pattern aligns with broader trends documented across implementation science. Recent analyses have noted that despite growing recognition of the importance of stakeholder engagement, even established implementation frameworks are often applied with limited depth, used retrospectively for data analysis rather than as organizing structures from inception [18], raising questions about whether participatory principles receive even less structural integration. The evolution of the field toward greater methodological standardization, while enabling cumulative knowledge-building, has raised concerns about whether this systematization comes at the cost of meaningful community partnership [19].

This does not imply that participatory principles have been wholly abandoned; indeed, CBPR appeared as a secondary framework in eight studies using implementation science as their primary approach. Rather, participatory methods appear to have shifted from organizing frameworks that structure entire evaluations to supplementary considerations within researcher-directed designs. Tambuyzer and colleagues’ typology of participation levels is instructive here: participation can range from informing and consulting stakeholders to genuine partnership and community control [20]. Our findings suggest that contemporary process evaluations may have moved down this ladder, from partnership toward consultation, even while maintaining the language of engagement.

The change is thus one of foregrounding and framing, not wholesale displacement. An alternative interpretation, however, deserves consideration: participatory approaches may not have disappeared so much as been absorbed into other frameworks or relabeled. What counted as “participatory research” in 2008 may now be described using implementation science terminology, with community advisory boards framed as “stakeholder engagement” and co-design processes labeled “adaptation.” Recent work on participatory research ethics suggests that definitions and interpretations of participation have themselves evolved over time, complicating direct comparison across eras [21]. Our data cannot fully adjudicate between genuine decline and terminological absorption, though the complete absence of CBPR, CPPR, and related frameworks as primary organizing structures after 2015 suggests the shift is more than semantic.

The temporal coincidence between this shift and influential methodological guidance warrants careful consideration. The 2015 MRC process evaluation guidance [7] and the subsequent updated framework for complex interventions [6] have been highly influential. These documents, while not dismissing participation, emphasize systematic examination of implementation fidelity, dose, reach, and mechanisms, constructs that align closely with implementation science frameworks. The guidance may have contributed to legitimizing certain approaches while implicitly signaling that others were less methodologically rigorous. This interpretation aligns with analyses of how authoritative guidance documents shape research practice, creating intellectual environments in which particular frameworks appear most ‘appropriate’ [22].

The subsequent decade saw the rapid ascendancy of implementation science frameworks. This shift coincided with the institutionalization of implementation science as a distinct field, including the establishment of dedicated journals such as Implementation Science in 2006 [14], dedicated funding mechanisms, and training programs [15]. Frameworks like CFIR and RE-AIM offered systematic, comprehensive approaches to examining implementation, with clearly specified domains and constructs that enabled structured data collection and cross-study comparison. The appeal of such systematization is evident in the consolidation observed: while early studies each used unique frameworks, later studies increasingly converged on a smaller set of established approaches.

This consolidation offers clear advantages. Shared frameworks enable cumulative knowledge building by providing common language and comparable constructs across studies. They offer methodological guidance for researchers new to process evaluation, reducing the burden of developing study-specific approaches. They also support the replication and synthesis that evidence-based practice requires. Moreover, the dominant frameworks themselves are evolving in response to the critiques our data implicitly raise: the updated CFIR [23] explicitly added equity-related constructs and recentered innovation recipients, reflecting growing recognition within implementation science that equity and stakeholder perspectives require structural integration rather than post-hoc consideration.

Yet this consolidation has also entailed losses. Participatory approaches embedded specific values about who should define evaluation questions, who should collect and interpret data, and whose perspectives should be centered. CBPR and related frameworks positioned community members not merely as information sources but as co-investigators with authority to shape research priorities and interpretations [24]. The disappearance of these frameworks from the process evaluation literature raises questions about whether and how community perspectives are being incorporated into contemporary evaluations.

This is not to suggest that current process evaluations ignore community perspectives; they do not. Studies using implementation science frameworks routinely collect data from community stakeholders. However, there is a distinction between incorporating community perspectives as data within a researcher-defined framework and positioning community members as partners in defining the evaluation framework itself. The shift toward systematization may represent a shift in epistemic authority (that is, who holds the power to define what counts as valid knowledge and how it should be generated) from communities to researchers, even as community voices continue to be heard.

### Framework fragmentation amid consolidation

A somewhat paradoxical finding is that substantial fragmentation persists despite categorical consolidation. While implementation science as a category dominates, no single framework accounts for more than 11% of studies (RE-AIM, n = 9). The field has converged on an approach, implementation science, without fully converging on specific frameworks within that approach.

This fragmentation creates challenges for synthesis and comparison. When studies use different frameworks, they examine different constructs and employ different measures, limiting the ability to compare findings or conduct meta-analyses. The proliferation of frameworks may also overwhelm researchers seeking guidance on framework selection, paradoxically undermining the goal of systematic, comparable evaluation.

This tension between consolidation and fragmentation mirrors broader debates in implementation science about framework proliferation. Nilsen’s influential taxonomy [8] identified over 60 frameworks, models, and theories in the field, and subsequent analyses suggest this number has continued to grow [15]. Some scholars have called for deliberate framework consolidation to enable synthesis, while others argue that theoretical diversity is necessary given the complexity of implementation contexts [10]. Our findings suggest that in community mental health specifically, the field has achieved categorical consolidation around implementation science while maintaining framework-level fragmentation, a pattern that may reflect researchers’ desire for theoretical legitimacy alongside uncertainty about which specific tools best fit their evaluation questions.

At the same time, framework diversity has value. Different frameworks illuminate different aspects of implementation. A field that converged entirely on a single framework would risk systematic blind spots. The challenge is to preserve the benefits of shared frameworks (cumulative knowledge, comparability, methodological guidance) while maintaining theoretical diversity sufficient to illuminate different aspects of implementation.

It is also worth noting that different framework categories may reflect different evaluation questions rather than merely different tools applied to the same questions. Participatory approaches and implementation science frameworks do not necessarily examine the same constructs or prioritize the same outcomes [25]. CBPR-guided evaluations may emphasize community empowerment, capacity building, and the quality of research partnerships, while CFIR-guided evaluations examine implementation determinants such as inner setting characteristics and intervention adaptability. The temporal shift we observe may therefore reflect a change not only in methodological preference but in what the field considers the appropriate focus of process evaluation, reflecting a shift in evaluation priorities alongside a shift in evaluation tools.

### Geographic concentration and equity implications

The geographic concentration of studies in North America (54%) and Europe (23%), with only 14% from low- and middle-income countries, raises significant concerns about the generalizability and equity implications of framework-based knowledge. This pattern is not unique to process evaluation; implementation science more broadly has been criticized for developing and validating frameworks primarily in high-income, Western contexts and then expecting these tools to transfer unproblematically to settings with fundamentally different health system structures, resource constraints, and cultural contexts [19].

Recent calls to prioritize health equity in implementation science are directly relevant here [19]. Frameworks developed in well-resourced settings may embed assumptions about infrastructure, workforce capacity, data systems, and community-researcher relationships that do not hold in LMIC contexts. Work on adapting implementation approaches for low-resource settings highlights the need for frameworks that account for different starting conditions rather than treating context as a barrier to overcome [26]. Our finding that all participatory approaches originated from North America is particularly notable: CBPR and related frameworks emerged from specific historical contexts of community organizing and social justice movements in American public health. Their absence from LMIC-based studies may reflect either genuine non-use or the application of local participatory traditions under different names, a question that warrants direct investigation.

Relatedly, the slower diffusion of implementation science frameworks into LMIC settings may reflect not only inequities in research infrastructure but also the relative scarcity of standardized, manualized community mental health interventions available for evaluation in these contexts. Where such interventions are still being established, the priorities of process evaluation may differ, centring on feasibility, acceptability, and cultural adaptation rather than fidelity to a predefined model. Researchers in these settings have often drawn on alternative or complementary approaches, including locally grounded participatory and community-based traditions, task-sharing and task-shifting models of the kind advanced through the World Health Organization’s Mental Health Gap Action Programme (mhGAP) [27], and culturally embedded evaluation practices that may not map neatly onto frameworks developed in high-income settings. Recognizing these alternatives, rather than treating implementation science frameworks as a universal default, may better support the development of contextually appropriate process evaluation in global mental health.

Emerging scholarship on equity-focused implementation science provides promising directions for addressing these gaps. Work on antiracism in implementation science [28] and equity-focused frameworks [29] suggests pathways for centering justice concerns within established implementation science structures, though whether such adaptations adequately address the distinct dynamics of community mental health evaluation remains to be tested.

Notably, the MRC Framework showed the opposite geographic pattern, appearing predominantly in European studies (4 of 6 MRC-guided studies, 67%), consistent with its British origins. As process evaluation expands globally, attention to developing and validating contextually appropriate frameworks should be a priority.

### Implications for future research and practice

For researchers designing process evaluations in community mental health, our typology offers a map for navigating framework selection. The choice of framework should be guided by the primary evaluation questions. Determinant frameworks like CFIR are appropriate when the goal is to identify factors affecting implementation success. Evaluation frameworks like RE-AIM are suited to assessing the reach, adoption, and sustainability of interventions. Process frameworks guide attention to implementation stages and activities. Program theory approaches like Theory of Change or Realist Evaluation are valuable when articulating and testing intervention logic.

Researchers should also consider explicitly justifying their framework choice and acknowledging what aspects of implementation may be less visible through their chosen lens. The common practice of referencing secondary frameworks suggests awareness that single frameworks may not capture all relevant dimensions. Making these choices transparent supports both interpretation of findings and cumulative learning across studies.

For methodologists and framework developers, our findings suggest several priorities. First, guidance is needed to help researchers select appropriate frameworks for their evaluation questions. Decision tools that map framework strengths to evaluation purposes could reduce both the burden on individual researchers and the fragmentation that comes from idiosyncratic choices. Second, attention to integrating participatory elements into implementation science frameworks could address the decline in community-engaged approaches without sacrificing systematization. Recent developments offer promising models: the updated MRC framework [6] gives greater attention to stakeholder involvement than its predecessors, and emerging work on equity-focused implementation explicitly centers community voice [19]. Frameworks for specific populations, including adolescents [30] and people with severe mental health conditions [31], have begun to articulate how participatory principles can be operationalized within implementation science structures. Third, the development and validation of frameworks appropriate for LMIC contexts should be prioritized to avoid the uncritical transfer of frameworks developed in high-income settings [26].

For funders and policymakers, our findings highlight the need to support both methodological development and capacity building. The concentration of process evaluation expertise in a small number of high-income countries limits the field’s ability to generate globally relevant knowledge. Supporting LMIC-based researchers to conduct and to lead process evaluations using contextually appropriate frameworks should be a priority.

### Strengths and limitations

This review has several strengths. We conducted comprehensive searches across multiple databases covering nearly two decades of literature. Our typology was developed inductively from the data, ensuring it reflects actual framework use rather than a priori categories. The inclusion of protocols and theoretical papers alongside empirical studies provides a complete picture of the methodological landscape. Temporal analysis revealed patterns that, while consistent with trends noted in broader implementation science literature, have not been systematically documented specifically within community mental health process evaluation.

Several limitations should be acknowledged. Screening was conducted primarily by the first author with secondary verification by the second author rather than fully independent dual screening, which may have introduced selection bias; the use of clearly operationalized inclusion criteria and consensus discussion of uncertain cases aimed to mitigate this risk. Similarly, data extraction was performed by the first author with regular review by the second author rather than independent dual extraction. Our search was limited to English-language publications, which may have contributed to the observed geographic concentration. The categorization of frameworks required judgment, particularly for frameworks that span multiple traditions; while we documented our decisions and reached consensus through discussion, others might categorize some frameworks differently. We assessed framework use as reported by authors rather than the quality or fidelity of framework application; a study claiming to use CFIR may have engaged with the framework superficially or comprehensively. Our search terms may have missed process evaluations conducted under other labels, such as formative evaluation or quality improvement, though we believe we captured the core literature. Additionally, our key finding regarding the temporal shift in participatory approaches rests on a relatively small number of studies (n = 5 in the early period), and while the pattern is striking, readers should interpret claims about paradigm shift with appropriate caution. The finding that participatory approaches continued to appear as secondary frameworks in later studies suggests the shift may be one of repositioning rather than disappearance, a more nuanced pattern than absolute numbers alone convey. Finally, scoping review methodology does not include quality assessment, limiting conclusions about the rigor of individual studies.

### Future research directions

Our findings point to several directions for future research. First, empirical comparison of what different frameworks reveal about the same implementation context would inform framework selection guidance. Second, detailed examination of process evaluation components (what aspects of implementation are actually examined across studies and how this relates to framework choice) would complement the present focus on framework use. We have extracted these data and will report them separately. Third, development and evaluation of hybrid approaches that integrate participatory principles with implementation science systematization could address the gaps our analysis has identified. Fourth, research on framework use in LMIC contexts, including development of contextually appropriate frameworks, is needed to support global mental health implementation.

## Conclusions

This scoping review reveals a field that has undergone substantial transformation over two decades. The landscape of process evaluation in community mental health has shifted from diverse, often participatory approaches toward consolidation around implementation science frameworks. This shift toward systematized, implementation science-driven evaluation has brought benefits in methodological rigor and comparability but has also coincided with the repositioning of approaches that centered community voice, from primary organizing frameworks to secondary considerations within researcher-directed designs.

The 54 distinct frameworks we identified, organized into 14 categories across 83 studies, reflect both consolidation and continued fragmentation. Implementation science frameworks, particularly CFIR and RE-AIM, have emerged as de facto standards, yet most frameworks appear in only one or two studies. This pattern suggests the field has adopted a shared paradigm without fully agreeing on specific theoretical tools.

Our typology offers researchers a map for navigating this complex landscape, organizing frameworks by theoretical orientation and function. However, a map is not a guide for selection. The field would benefit from explicit guidance to help researchers match frameworks to evaluation purposes, and from continued attention to integrating the values of community participation with the systematization that implementation science provides.

As community mental health interventions proliferate globally and mental health assumes greater priority in health policy, the quality and relevance of process evaluation becomes increasingly consequential. Implementation science itself is increasingly recognizing that equity, context-sensitivity, and meaningful stakeholder partnership are not optional additions but essential features of rigorous implementation research [15,19]. Ensuring that evaluation approaches in community mental health embody both scientific rigor and these participatory values, moving, perhaps, toward what might be called ‘systematized participation’, remains a critical challenge and opportunity for the field.

## Supporting information

S1 File Search strategies for PubMed, Web of Science, and EBSCOhost databases. (DOCX)

S2 File Complete list of included studies (N = 83). (DOCX)

S1 Checklist PRISMA-ScR checklist. (DOCX)
