# Supplementary material for: From participation to systematization: A scoping review of theoretical frameworks guiding process evaluations in community mental health interventions
Source: PLoS One. 2026 Jul 28;21(7):e0354732. doi: 10.1371/journal.pone.0354732 (PMC13411908; doi:10.1371/journal.pone.0354732)
Supplement: S1 File — (DOCX) [file pone.0354732.s001.docx]

**Supplementary File 1**

***Search Strategies***

Searches were conducted across three databases from January 2006 to April 2025. The search strategy combined three concept blocks: (1) process evaluation and implementation terminology, (2) community settings, and (3) mental health conditions. Database-specific syntax was adapted for each platform as detailed below.

| **Database** | **Date Searched** | **Search Fields** |
| --- | --- | --- |
| PubMed | April 2025 | Title/Abstract |
| Web of Science Core Collection | April 2025 | Topic (TS) |
| EBSCOhost (APA PsycArticles + Academic Search Ultimate) | April 2025 | Abstract (AB) |

**PubMed**

("process evaluation"[Title/Abstract] OR "implementation framework"[Title/Abstract] OR "theoretical framework"[Title/Abstract] OR "program evaluation"[Title/Abstract] OR "implementation science"[Title/Abstract])

AND

("community mental health"[Title/Abstract] OR "community-based"[Title/Abstract] OR "population-based"[Title/Abstract] OR "public mental health"[Title/Abstract])

AND

("mental health"[Title/Abstract] OR "depression"[Title/Abstract] OR "suicide prevention"[Title/Abstract] OR "suicidal behavior"[Title/Abstract])

*Limits: Publication date 2006/01/01–2025/04/30; English*

**Web of Science Core Collection**

TS=("process evaluation" OR "implementation framework" OR "theoretical framework" OR "program evaluation" OR "implementation science")

AND

TS=("community mental health" OR "community-based" OR "population-based" OR "public mental health")

AND

TS=("mental health" OR "depression" OR "suicide prevention" OR "suicidal behavior")

*Limits: Publication years 2006–2025; Language: English; Document types: Article, Review Article, Early Access*

**EBSCOhost (APA PsycArticles + Academic Search Ultimate)**

AB("process evaluation" OR "implementation framework" OR "theoretical framework" OR "program evaluation" OR "implementation science")

AND

AB("community mental health" OR "community-based" OR "population-based" OR "public mental health")

AND

AB("mental health" OR "depression" OR "suicide prevention" OR "suicidal behavior")

*Limits: Published date 2006–2025; Language: English; Peer-reviewed*

***Note.*** *All searches were limited to English-language, peer-reviewed journal articles published between January 2006 and April 2025. Results were imported into Rayyan systematic review software for duplicate removal and screening.*
