# Supplementary material for: From participation to systematization: A scoping review of theoretical frameworks guiding process evaluations in community mental health interventions
Source: PLoS One. 2026 Jul 28;21(7):e0354732. doi: 10.1371/journal.pone.0354732 (PMC13411908; doi:10.1371/journal.pone.0354732)
Supplement: S2 File — (DOCX) [file pone.0354732.s002.docx]

**Supplementary File 2**

***Characteristics of Included Studies (N = 83)***

| **First Author** | **Year** | **Domain** | **Region** | **Primary Framework** | **Framework Category** |
| --- | --- | --- | --- | --- | --- |
| Allison-Burbank | 2025 | Suicide prevention | North America | Indigenous Connectedness Framework | Hybrid/Multiple frameworks |
| Alvarez-Monjaras | 2023 | Broader mental health | Europe | Donabedian structure-process-outcome | Quality/Fidelity |
| Ball | 2025 | Broader mental health | Multi-region | Multiple - scoping review of TMFs | Review (meta-level) |
| Baxter | 2020 | Broader mental health | Europe | COM-B | Behavior change theory |
| Becker-Haimes | 2021 | Depression-focused | North America | Theory of Planned Behavior | Behavior change theory |
| Beidas | 2014 | Broader mental health | North America | Organizational Social Context (OSC) | Other (non-IS framework) |
| Bluthenthal | 2006 | Depression-focused | North America | HAAF community mobilization | Participatory approaches |
| Burroughs | 2019 | Depression-focused | Europe | Behavioral activation (intervention) | Other (non-IS framework) |
| Byng | 2008 | Broader mental health | Europe | Realistic Evaluation | Program theory |
| Cabassa | 2014 | Broader mental health | North America | Collaborative intervention planning | Participatory approaches |
| Cappella | 2011 | Broader mental health | North America | CBPR | Participatory approaches |
| Cheron | 2019 | Broader mental health | North America | Active Implementation Framework (AIF)/NIRN | Implementation science - process |
| Cohen | 2020 | Broader mental health | North America | Implementation Stages (Fixsen) | Implementation science - process |
| Cuxart-Graell | 2024 | Broader mental health | Europe | MRC Framework | Process evaluation specific |
| Doukani | 2021 | Depression-focused | Europe | Weiner's ORIC | Implementation science - determinants |
| Eastwood | 2019 | Broader mental health | Asia-Pacific | MRC Framework | Process evaluation specific |
| Eaton | 2018 | Broader mental health | Middle East/Africa | Theory of Change | Program theory |
| Ellis | 2023 | Suicide prevention | Asia-Pacific | Berg and Aase resilience framework | Other (non-IS framework) |
| Fortuna | 2023 | Broader mental health | North America | CFIR | Implementation science - determinants |
| Fung | 2021 | Broader mental health | Asia-Pacific | RE-AIM | Implementation science - evaluation |
| Gleacher | 2016 | Broader mental health | North America | Aarons et al. implementation framework | Implementation science - determinants |
| Goto | 2024 | Broader mental health | North America | Theory of Planned Behavior | Behavior change theory |
| Greene | 2024 | Broader mental health | Latin America | RE-AIM | Implementation science - evaluation |
| Greenley | 2024 | Broader mental health | Middle East/Africa | MRC Framework | Process evaluation specific |
| Hadjistavropoulos | 2017 | Depression-focused | North America | CFIR | Implementation science - determinants |
| Hanbury | 2011 | Suicide prevention | Europe | Theory of Planned Behavior | Behavior change theory |
| Hanlon | 2022 | Suicide prevention | Europe | Suicide theories integration (IPT/CAMS/IMV) | Other (non-IS framework) |
| Harker Roa | 2023 | Broader mental health | Latin America | Proctor et al. implementation outcomes | Implementation science - evaluation |
| Harris | 2016 | Suicide prevention | Europe | MRC Framework | Process evaluation specific |
| Hegerl | 2009 | Suicide prevention | Europe | EAAD 4-level (+5th level) | Public health/Prevention model |
| Hegerl | 2019 | Suicide prevention | Europe | MRC Framework | Process evaluation specific |
| Hickey | 2018 | Broader mental health | Europe | Implementation Stages (Fixsen) | Implementation science - process |
| Hoffacker | 2022 | Broader mental health | North America | Theory of Planned Behavior | Behavior change theory |
| Hudson | 2019 | Broader mental health | Europe | CFIR | Implementation science - determinants |
| Kassab | 2022 | Depression-focused | North America | Framework for Dissemination | Implementation science - process |
| Khodyakov | 2009 | Depression-focused | North America | CPPR (Community-Partnered Participatory Research) | Participatory approaches |
| Kimber | 2019 | Broader mental health | North America | None explicit (IS general) | None explicit |
| Kutash | 2012 | Broader mental health | North America | Five-component fidelity model | Quality/Fidelity |
| Kwan | 2019 | Broader mental health | Multi-region | RE-AIM | Implementation science - evaluation |
| Lai | 2020 | Suicide prevention | Asia-Pacific | Public health approach (3-level) | Public health/Prevention model |
| Lang | 2015 | Broader mental health | North America | Breakthrough Series Collaborative | Implementation science - process |
| Le | 2023 | Broader mental health | Latin America | CFIR | Implementation science - determinants |
| Leamy | 2014 | Broader mental health | Europe | REFOCUS intervention model | Other (non-IS framework) |
| Lewis | 2019 | Broader mental health | North America | None explicit (IS general) | None explicit |
| Lewis 2018a | 2018 | Depression-focused | North America | Framework for Dissemination | Implementation science - process |
| Lewis 2018b | 2018 | Broader mental health | North America | Framework for Dissemination | Implementation science - process |
| Lyon | 2018 | Broader mental health | North America | EPIS | Implementation science - determinants |
| McNeish | 2022 | Broader mental health | North America | NIRN Active Implementation Framework | Implementation science - process |
| McNeish Taormina | 2023 | Broader mental health | North America | BC4 model (study-developed) | Study-developed |
| Mendenhall | 2014 | Broader mental health | North America | Theory of Change (systems of care) | Program theory |
| Mercado | 2024 | Broader mental health | North America | RE-AIM | Implementation science - evaluation |
| Michie | 2007 | Broader mental health | Europe | 11 theoretical domains (TDF precursor) | Behavior change theory |
| Mignogna | 2023 | Depression-focused | North America | RE-AIM | Implementation science - evaluation |
| Mishara | 2023 | Suicide prevention | North America | Theory of Change | Program theory |
| Mora Ringle | 2024 | Broader mental health | North America | None explicit | None explicit |
| Munson | 2012 | Depression-focused | North America | Four-facet framework (study-developed) | Study-developed |
| Nakkash 2012a | 2012 | Broader mental health | Middle East/Africa | Ecological approach | Hybrid/Multiple frameworks |
| Nakkash 2012b | 2012 | Broader mental health | Middle East/Africa | Linnan & Steckler PE framework | Process evaluation specific |
| Nastasi | 2009 | Broader mental health | North America | CMMPE | Hybrid/Multiple frameworks |
| Ngo | 2024 | Broader mental health | North America | CFIR | Implementation science - determinants |
| Omer | 2016 | Broader mental health | Europe | RE-AIM | Implementation science - evaluation |
| Palmer | 2016 | Broader mental health | Asia-Pacific | RE-AIM | Implementation science - evaluation |
| Palmer Molina | 2024 | Depression-focused | North America | RE-AIM | Implementation science - evaluation |
| Pegg | 2021 | Broader mental health | North America | Modified grounded theory | None explicit |
| Petersen | 2011 | Broader mental health | Middle East/Africa | Common implementation framework | Implementation science - process |
| Priebe | 2017 | Broader mental health | Europe | DIALOG+ intervention framework | Other (non-IS framework) |
| Ritchie | 2016 | Broader mental health | North America | CFIR | Implementation science - determinants |
| Semrau | 2023 | Broader mental health | Multi-region | CFIR (updated) | Implementation science - determinants |
| Steinman | 2023 | Depression-focused | North America | MADI (Model for Adaptation Design and Impact) | Hybrid/Multiple frameworks |
| Stephens | 2014 | Broader mental health | North America | PRISM | Implementation science - determinants |
| Tewari | 2021 | Broader mental health | Asia-Pacific | Andersen's Behavioral Model | Health services/Utilization |
| Ungar | 2015 | Broader mental health | North America | Knowledge Mobilization (KMb) | Participatory approaches |
| Van Daele | 2012 | Depression-focused | Europe | Empowerment implementation (study-developed) | Study-developed |
| Vax | 2021 | Broader mental health | North America | Transtheoretical Model (TTM) | Behavior change theory |
| Weiss | 2021 | Broader mental health | North America | Population health approach | Public health/Prevention model |
| White | 2022 | Suicide prevention | North America | CFIR | Implementation science - determinants |
| Williams | 2019 | Broader mental health | Europe | MRC Framework | Process evaluation specific |
| Woltmann | 2023 | Broader mental health | North America | CFIR | Implementation science - determinants |
| Wood | 2024 | Suicide prevention | Asia-Pacific | RE-AIM | Implementation science - evaluation |
| Woodard | 2025 | Broader mental health | North America | Implementation Index framework | Quality/Fidelity |
| Woodward | 2023 | Suicide prevention | North America | ENGAGED for CHANGE | Hybrid/Multiple frameworks |
| Yusa | 2016 | Broader mental health | North America | Owen's evaluation use typology | Other (non-IS framework) |
| Zbukvic | 2022 | Suicide prevention | Asia-Pacific | EPIS | Implementation science - determinants |

***Note.*** *Studies sorted alphabetically by first author. Full references are available from the corresponding author on request.*
