# Supplementary material for: From participation to systematization: A scoping review of theoretical frameworks guiding process evaluations in community mental health interventions
Source: PLoS One. 2026 Jul 28;21(7):e0354732. doi: 10.1371/journal.pone.0354732 (PMC13411908; doi:10.1371/journal.pone.0354732)
Supplement: S1 Checklist — (DOCX) [file pone.0354732.s003.docx]

**S1 Checklist. Preferred Reporting Items for Systematic reviews and Meta-Analyses extension for Scoping Reviews (PRISMA-ScR) Checklist**

*Tricco AC, Lillie E, Zarin W, O’Brien KK, Colquhoun H, Levac D, et al. PRISMA Extension for Scoping Reviews (PRISMA-ScR): Checklist and Explanation. Ann Intern Med. 2018;169(7):467–473.*

| **SECTION** | **ITEM** | **PRISMA-ScR CHECKLIST ITEM** | **CHECKLIST ITEM DESCRIPTION** | **REPORTED ON PAGE #/ SECTION** |
| --- | --- | --- | --- | --- |
| **TITLE** | 1 | Title | Identify the report as a scoping review. | Title page, p. 1 |
| **ABSTRACT** | 2 | Structured summary | Provide a structured summary that includes (as applicable): background, objectives, eligibility criteria, sources of evidence, charting methods, results, and conclusions. | Abstract, pp. 2–3 |
| **INTRODUCTION** |  |  |  |  |
|  | 3 | Rationale | Describe the rationale for the review in the context of what is already known. Explain why the review questions/objectives lend themselves to a scoping review approach. | Introduction, paragraphs 1–4 |
|  | 4 | Objectives | Provide an explicit statement of the questions and objectives being addressed with reference to their key elements (e.g., population or participants, concepts, and context) or other relevant key elements used to conceptualize the review questions and/or objectives. | Introduction, final paragraph (objectives 1–4) |
| **METHODS** |  |  |  |  |
|  | 5 | Protocol and registration | Indicate whether a review protocol exists; state if and where it can be accessed (e.g., a Web address); and if available, provide registration information, including the registration number. | Methods – Design: "This review was not prospectively registered; however, the research questions and analytical approach were established prior to data extraction." |
|  | 6 | Eligibility criteria | Specify characteristics of the sources of evidence used as eligibility criteria (e.g., years considered, language, and publication status), and provide a rationale. | Methods – Eligibility criteria |
|  | 7 | Information sources | Describe all information sources in the search (e.g., databases with dates of coverage and contact with authors to identify additional sources), as well as the date the most recent search was executed. | Methods – Information sources and search strategy; S1 File |
|  | 8 | Search | Present the full electronic search strategy for at least 1 database, including any limits used, such that it could be repeated. | S1 File (complete search strategies for all 3 databases) |
|  | 9 | Selection of sources of evidence | State the process for selecting sources of evidence (i.e., screening and eligibility) included in the scoping review. | Methods – Selection process |
|  | 10 | Data charting process | Describe the methods of charting data from the included sources of evidence (e.g., calibrated forms or forms that have been tested by the team before their use, and whether data charting was done independently or in duplicate) and any processes for obtaining and confirming data from investigators. | Methods – Data extraction |
|  | 11 | Data items | List and define all variables for which data were sought and any assumptions and simplifications made. | Methods – Data extraction (lists all extracted variables) |
|  | 12 | Critical appraisal of individual sources of evidence | If done, provide a rationale for conducting a critical appraisal of included sources of evidence; describe the methods used and how this information was used in any data synthesis (if applicable). | Not applicable – consistent with scoping review methodology, quality assessment was not conducted (acknowledged in Strengths and limitations) |
|  | 13 | Synthesis of results | Describe the methods of handling and summarizing the data that were charted. | Methods – Framework typology development; Data synthesis and analysis |
| **RESULTS** |  |  |  |  |
|  | 14 | Selection of sources of evidence | Give numbers of sources of evidence screened, assessed for eligibility, and included in the review, with reasons for exclusions at each stage, ideally using a flow diagram. | Results – Study selection; Fig 1 |
|  | 15 | Characteristics of sources of evidence | For each source of evidence, present characteristics for which data were charted and provide the citations. | Results – Study characteristics (Table 1); S2 File |
|  | 16 | Critical appraisal within sources of evidence | If done, present data on critical appraisal of included sources of evidence (see item 12). | Not applicable |
|  | 17 | Results of individual sources of evidence | For each included source of evidence, present the relevant data that were charted that relate to the review questions and objectives. | S2 File (complete characteristics of all 83 studies) |
|  | 18 | Synthesis of results | Summarize and/or present the charting results as they relate to the review questions and objectives. | Results – Framework typology through Framework use by domain (Tables 2–5; Figs 1–2) |
| **DISCUSSION** |  |  |  |  |
|  | 19 | Summary of evidence | Summarize the main results (including an overview of concepts, themes, and types of evidence available), link to the review questions and objectives, and consider the relevance to key groups. | Discussion – Summary of principal findings |
|  | 20 | Limitations | Discuss the limitations of the scoping review process. | Discussion – Strengths and limitations |
|  | 21 | Conclusions | Provide a general interpretation of the results with respect to the review questions and objectives, as well as potential implications and/or next steps. | Conclusions; Discussion – Implications for future research and practice; Future research directions |
| **FUNDING** | 22 | Funding | Describe sources of funding for the included sources of evidence, as well as sources of funding for the scoping review. Describe the role of the funders of the scoping review. | Financial Disclosure section of the submission system (not included in manuscript per journal policy). |
