# Supplementary material for: From participation to systematization: A scoping review of theoretical frameworks guiding process evaluations in community mental health interventions
Source: PLoS One. 2026 Jul 28;21(7):e0354732. doi: 10.1371/journal.pone.0354732 (PMC13411908; doi:10.1371/journal.pone.0354732)
Supplement: Attachment — Submitted filename: Response_to_Reviewers.docx [file pone.0354732.s005.docx]

**Response to Reviewers**

Manuscript ID: PONE-D-26-09912

*Title: From participation to systematization: A scoping review of theoretical frameworks guiding process evaluations in community mental health interventions*

Dear Dr. Baker,

Thank you for the opportunity to revise our manuscript, and please pass on our thanks to both reviewers for their careful and constructive reading. We were pleased that both reviewers found the work technically sound, appropriately analyzed, and clearly written. We have addressed each point below. Reviewer comments are shown in italics, followed by our responses. All changes are visible in the marked-up manuscript and have been incorporated into the clean version.

**Journal Requirements**

**1. Style requirements and file naming.** We have reviewed the manuscript against the PLOS ONE style templates for the title page and main body and have named all submission files in line with PLOS ONE conventions.

**2. Funding statement.** We have removed all funding-related text from the manuscript. The Acknowledgments section had contained only a brief statement of the first author’s doctoral context together with the grant information; as funding details may not appear in the manuscript file, the section no longer served a purpose and has been removed in full. We would like the Funding Statement to be updated to the following:

*RM received funding through the Cross-Sectoral Mobility Measure (SekMo) Knowledge Transfer Doctoral Programme, financed by the European Regional Development Fund (project no. 2021-2027.1.01.24-0639). This work was also conducted within the MENTBEST project, funded by the European Union under Horizon Europe (grant agreement No 101080651). The funders had no role in study design, data collection and analysis, decision to publish, or preparation of the manuscript.*

**3. Citation of recommended works.** No specific previously published works were recommended for citation. In responding to Reviewer 2, we evaluated the relevant literature and added one reference (the WHO Mental Health Gap Action Programme, mhGAP) where it directly supported a new point in the Discussion.

**4. Reference list.** We have checked the reference list for completeness and accuracy and confirm that none of the cited works has been retracted. One new reference has been added (now reference 31); no references were removed.

**Figures.** In line with the figure guidance in the decision letter, both figures have been re-exported to meet the PLOS technical specifications (TIFF, RGB, 300 dpi, within the required pixel dimensions) and are uploaded as individual files named Fig1.tif and Fig2.tif.

**Reviewer 1**

*I have read the manuscript well and I am satisfied with the work that the authors have done with this manuscript, it has been written and presented in an intelligible standard English, and hence I have no additional questions or comments.*

We thank Reviewer 1 for the positive assessment. No changes were requested, and none were required in response to this review.

**Reviewer 2**

*This systematic review focuses on an interesting area to guide process evaluation of community based mental health interventions.*

We thank Reviewer 2 for the encouraging assessment and for two helpful points, which we address in turn.

**Comment 2.1.**

*In page 15, did the interventions include alcohol and substance use disorder including co-occurring HIV / HBV? Which age groups were considered in inclusion? Only adults or adolescents also?*

We appreciate this request to clarify the scope of included interventions. To make our eligibility criteria explicit, we have added the following clarification to the Methods (Eligibility criteria):

*“No age restrictions were applied, so eligible interventions spanned all age groups, from adolescents in school-based settings to adults in workplace and community settings.”*

To address the reviewer’s specific questions directly: no age limits were applied, so both adolescents and adults are represented in the corpus (school-based interventions, for example, frequently targeted adolescents). Substance use disorder was not one of our three target domains (broader mental health, depression, and suicide prevention), although interventions addressing co-occurring common mental disorders were eligible under the broader mental health domain. Interventions defined primarily by physical comorbidities, such as co-occurring HIV or hepatitis B, fell outside the mental health focus of this review and were therefore not included. We have kept the manuscript addition concise, clarifying the age range of eligible populations, since that was the parameter not previously stated; the substance use and comorbidity points are addressed here rather than in the manuscript text.

**Comment 2.2.**

*The authors have identified a shift to implementation science in economically advanced nations. It may be noted that in LMICs there is still a gap in availability of standardized community based mental health interventions. Therefore, the progress of implementation science is slower in LMICs. In this context some discussion on alternatives to implementation science in LMICs will be more useful.*

This is a valuable observation, and we agree that the relative scarcity of standardized community mental health interventions in many LMIC settings is itself part of the explanation for the slower uptake of implementation science frameworks there. We have added a paragraph to the Discussion (Geographic concentration and equity implications) that develops this point and discusses alternative and complementary approaches:

*“Relatedly, the slower diffusion of implementation science frameworks into LMIC settings may reflect not only inequities in research infrastructure but also the relative scarcity of standardized, manualized community mental health interventions available for evaluation in these contexts. Where such interventions are still being established, the priorities of process evaluation may differ, centring on feasibility, acceptability, and cultural adaptation rather than fidelity to a predefined model. Researchers in these settings have often drawn on alternative or complementary approaches, including locally grounded participatory and community-based traditions, task-sharing and task-shifting models of the kind advanced through the World Health Organization’s Mental Health Gap Action Programme (mhGAP), and culturally embedded evaluation practices that may not map neatly onto frameworks developed in high-income settings. Recognizing these alternatives, rather than treating implementation science frameworks as a universal default, may better support the development of contextually appropriate process evaluation in global mental health.”*

We have added a supporting reference to the WHO mhGAP programme (reference 31) to accompany this addition.

We hope these revisions satisfactorily address the points raised. Thank you again for your consideration.

Sincerely,

Rainer Mere, on behalf of all authors
