# Supplementary material for: From participation to systematization: A scoping review of theoretical frameworks guiding process evaluations in community mental health interventions
Source: PLoS One. 2026 Jul 28;21(7):e0354732. doi: 10.1371/journal.pone.0354732 (PMC13411908; doi:10.1371/journal.pone.0354732)
Supplement: Peer review history

# Peer review: Decision Letter 0

**DOI:** 10.1371/journal.pone.0354732.r001

**Authors:** Rogis Baker

26 May 2026

PONE-D-26-09912

From participation to systematization: A scoping review of theoretical frameworks guiding process evaluations in community mental health interventions

PLOS One

Dear Dr. Mere,

Thank you for submitting your manuscript to PLOS ONE. After careful consideration, we feel that it has merit but does not fully meet PLOS ONE’s publication criteria as it currently stands. Therefore, we invite you to submit a revised version of the manuscript that addresses the points raised during the review process.

Please submit your revised manuscript by Jul 10 2026 11:59PM. If you will need more time than this to complete your revisions, please reply to this message or contact the journal office at plosone@plos.org. When you're ready to submit your revision, log on to https://www.editorialmanager.com/pone/ and select the 'Submissions Needing Revision' folder to locate your manuscript file.

Please include the following items when submitting your revised manuscript:

- A letter that responds to each point raised by the academic editor and reviewer(s). You should upload this letter as a separate file labeled 'Response to Reviewers'.
- A marked-up copy of your manuscript that highlights changes made to the original version. You should upload this as a separate file labeled 'Revised Manuscript with Track Changes'.
- An unmarked version of your revised paper without tracked changes. You should upload this as a separate file labeled 'Manuscript'.

If you would like to make changes to your financial disclosure, please include your updated statement in your cover letter. Guidelines for resubmitting your figure files are available below the reviewer comments at the end of this letter.

If applicable, we recommend that you deposit your laboratory protocols in protocols.io to enhance the reproducibility of your results. Protocols.io assigns your protocol its own identifier (DOI) so that it can be cited independently in the future. For instructions see: https://journals.plos.org/plosone/s/submission-guidelines#loc-laboratory-protocols. Additionally, PLOS ONE offers an option for publishing peer-reviewed Lab Protocol articles, which describe protocols hosted on protocols.io. Read more information on sharing protocols at https://plos.org/protocols?utm_medium=editorial-email&utm_source=authorletters&utm_campaign=protocols.

As the corresponding author, your ORCID iD is verified in the submission system and will appear in the published article. PLOS supports the use of ORCID, and we encourage all coauthors to register for an ORCID iD and use it as well. Please encourage your coauthors to verify their ORCID iD within the submission system before final acceptance, as unverified ORCID iDs will not appear in the published article. *Only*  the individual author can complete the verification step; PLOS staff *cannot*  verify ORCID iDs on behalf of authors.

We look forward to receiving your revised manuscript.

Kind regards,

Rogis Baker, Ph.D

Academic Editor

PLOS One

**Journal Requirements:**

When submitting your revision, we need you to address these additional requirements.

1. Please ensure that your manuscript meets PLOS ONE's style requirements, including those for file naming. The PLOS ONE style templates can be found at

https://journals.plos.org/plosone/s/file?id=wjVg/PLOSOne_formatting_sample_main_body.pdf and

https://journals.plos.org/plosone/s/file?id=ba62/PLOSOne_formatting_sample_title_authors_affiliations.pdf

2. Thank you for stating the following in the Acknowledgments Section of your manuscript:

“This scoping review was conducted as part of the first author’s doctoral research at Tallinn University, carried out in the context of the MENTBEST project (EU Horizon Europe, grant agreement No 101080651).”

We note that you have provided funding information that is not currently declared in your Funding Statement. However, funding information should not appear in the Acknowledgments section or other areas of your manuscript. We will only publish funding information present in the Funding Statement section of the online submission form.

Please remove any funding-related text from the manuscript and let us know how you would like to update your Funding Statement. Currently, your Funding Statement reads as follows:

“RM received funding through the Cross-Sectoral Mobility Measure (SekMo) Knowledge Transfer Doctoral Programme, financed by the European Regional Development Fund (project no. 2021-2027.1.01.24-0639). The funders had no role in study design, data collection and analysis, decision to publish, or preparation of the manuscript.”

Please include your amended statements within your cover letter; we will change the online submission form on your behalf.

3. If the reviewer comments include a recommendation to cite specific previously published works, please review and evaluate these publications to determine whether they are relevant and should be cited. There is no requirement to cite these works unless the editor has indicated otherwise.

4. Please review your reference list to ensure that it is complete and correct. If you have cited papers that have been retracted, please include the rationale for doing so in the manuscript text, or remove these references and replace them with relevant current references. Any changes to the reference list should be mentioned in the rebuttal letter that accompanies your revised manuscript. If you need to cite a retracted article, indicate the article’s retracted status in the References list and also include a citation and full reference for the retraction notice.

[Note: HTML markup is below. Please do not edit.]

Reviewers' comments:

Reviewer's Responses to Questions

**Comments to the Author**

1. Is the manuscript technically sound, and do the data support the conclusions?

The manuscript must describe a technically sound piece of scientific research with data that supports the conclusions. Experiments must have been conducted rigorously, with appropriate controls, replication, and sample sizes. The conclusions must be drawn appropriately based on the data presented.

Reviewer #1: Yes

Reviewer #2: Yes

**********

2. Has the statistical analysis been performed appropriately and rigorously?

Reviewer #1: Yes

Reviewer #2: Yes

**********

3. Have the authors made all data underlying the findings in their manuscript fully available?

The PLOS Data policy requires authors to make all data underlying the findings described in their manuscript fully available without restriction, with rare exception (please refer to the Data Availability Statement in the manuscript PDF file). The data should be provided as part of the manuscript or its supporting information, or deposited to a public repository. For example, in addition to summary statistics, the data points behind means, medians and variance measures should be available. If there are restrictions on publicly sharing data—e.g. participant privacy or use of data from a third party—those must be specified.

Reviewer #1: Yes

Reviewer #2: Yes

**********

4. Is the manuscript presented in an intelligible fashion and written in standard English?

PLOS ONE does not copyedit accepted manuscripts, so the language in submitted articles must be clear, correct, and unambiguous. Any typographical or grammatical errors should be corrected at revision, so please note any specific errors here.

Reviewer #1: Yes

Reviewer #2: Yes

**********

5. Review Comments to the Author

Please use the space provided to explain your answers to the questions above. You may also include additional comments for the author, including concerns about dual publication, research ethics, or publication ethics. (Please upload your review as an attachment if it exceeds 20,000 characters)

Reviewer #1: I have read the manuscript well and I am satisfied with the work that the authors have done with this manuscript, it has been written and presented in an intelligible standard english, and hence I have no adittional questions or comments.

Reviewer #2: This systematic review focuses on an interesting area to guide process evaluation of community based mental health interventions.

In page 15, did the interventions include alcohol and substance use disorder including co-occurring HIV / HBV? Which age groups were considered in inclusion? Only adults or adolescents also?

The authors have identified a shift to implementation science in economically advanced nations. It may be noted that in LMICs there is still a gap in availability of standardized community based mental health interventions. Therefore, the progress of implementation science is slower in LMICs. In this context some discussion on alternatives to implementation science in LMICs will be more useful.

**********

6. PLOS authors have the option to publish the peer review history of their article (what does this mean?). If published, this will include your full peer review and any attached files.

If you choose “no”, your identity will remain anonymous but your review may still be made public.

**Do you want your identity to be public for this peer review?**  For information about this choice, including consent withdrawal, please see our Privacy Policy.

Reviewer #1: No

Reviewer #2: **Yes:**  Amit Chakrabarti

**********

[NOTE: If reviewer comments were submitted as an attachment file, they will be attached to this email and accessible via the submission site. Please log into your account, locate the manuscript record, and check for the action link "View Attachments". If this link does not appear, there are no attachment files.]

To ensure your figures meet our technical requirements, please review our figure guidelines: https://journals.plos.org/plosone/s/figures

You may also use PLOS’s free figure tool, NAAS, to help you prepare publication quality figures: https://journals.plos.org/plosone/s/figures#loc-tools-for-figure-preparation.

NAAS will assess whether your figures meet our technical requirements by comparing each figure against our figure specifications.

# Author comment: Author response to Decision Letter 1

**DOI:** 10.1371/journal.pone.0354732.r002

15 Jun 2026

Response to Reviewers

Manuscript ID: PONE-D-26-09912

Title: From participation to systematization: A scoping review of theoretical frameworks guiding process evaluations in community mental health interventions

Dear Dr. Baker,

Thank you for the opportunity to revise our manuscript, and please pass on our thanks to both reviewers for their careful and constructive reading. We were pleased that both reviewers found the work technically sound, appropriately analyzed, and clearly written. We have addressed each point below. All changes are visible in the marked-up manuscript and have been incorporated into the clean version.

Journal Requirements

1. Style requirements and file naming. We have reviewed the manuscript against the PLOS ONE style templates for the title page and main body and have named all submission files in line with PLOS ONE conventions.

2. Funding statement. We have removed all funding-related text from the manuscript. The Acknowledgments section had contained only a brief statement of the first author’s doctoral context together with the grant information; as funding details may not appear in the manuscript file, the section no longer served a purpose and has been removed in full. We would like the Funding Statement to be updated to the following:

RM received funding through the Cross-Sectoral Mobility Measure (SekMo) Knowledge Transfer Doctoral Programme, financed by the European Regional Development Fund (project no. 2021-2027.1.01.24-0639). This work was also conducted within the MENTBEST project, funded by the European Union under Horizon Europe (grant agreement No 101080651). The funders had no role in study design, data collection and analysis, decision to publish, or preparation of the manuscript.

3. Citation of recommended works. No specific previously published works were recommended for citation. In responding to Reviewer 2, we evaluated the relevant literature and added one reference (the WHO Mental Health Gap Action Programme, mhGAP) where it directly supported a new point in the Discussion.

4. Reference list. We have checked the reference list for completeness and accuracy and confirm that none of the cited works has been retracted. One new reference has been added (now reference 31); no references were removed.

Figures. In line with the figure guidance in the decision letter, both figures have been re-exported to meet the PLOS technical specifications (TIFF, RGB, 300 dpi, within the required pixel dimensions) and are uploaded as individual files named Fig1.tif and Fig2.tif.

Reviewer 1

I have read the manuscript well and I am satisfied with the work that the authors have done with this manuscript, it has been written and presented in an intelligible standard English, and hence I have no additional questions or comments.

We thank Reviewer 1 for the positive assessment. No changes were requested, and none were required in response to this review.

Reviewer 2

This systematic review focuses on an interesting area to guide process evaluation of community based mental health interventions.

We thank Reviewer 2 for the encouraging assessment and for two helpful points, which we address in turn.

Comment 2.1.

In page 15, did the interventions include alcohol and substance use disorder including co-occurring HIV / HBV? Which age groups were considered in inclusion? Only adults or adolescents also?

We appreciate this request to clarify the scope of included interventions. To make our eligibility criteria explicit, we have added the following clarification to the Methods (Eligibility criteria):

“No age restrictions were applied, so eligible interventions spanned all age groups, from adolescents in school-based settings to adults in workplace and community settings.”

To address the reviewer’s specific questions directly: no age limits were applied, so both adolescents and adults are represented in the corpus (school-based interventions, for example, frequently targeted adolescents). Substance use disorder was not one of our three target domains (broader mental health, depression, and suicide prevention), although interventions addressing co-occurring common mental disorders were eligible under the broader mental health domain. Interventions defined primarily by physical comorbidities, such as co-occurring HIV or hepatitis B, fell outside the mental health focus of this review and were therefore not included. We have kept the manuscript addition concise, clarifying the age range of eligible populations, since that was the parameter not previously stated; the substance use and comorbidity points are addressed here rather than in the manuscript text.

Comment 2.2.

The authors have identified a shift to implementation science in economically advanced nations. It may be noted that in LMICs there is still a gap in availability of standardized community based mental health interventions. Therefore, the progress of implementation science is slower in LMICs. In this context some discussion on alternatives to implementation science in LMICs will be more useful.

This is a valuable observation, and we agree that the relative scarcity of standardized community mental health interventions in many LMIC settings is itself part of the explanation for the slower uptake of implementation science frameworks there. We have added a paragraph to the Discussion (Geographic concentration and equity implications) that develops this point and discusses alternative and complementary approaches:

“Relatedly, the slower diffusion of implementation science frameworks into LMIC settings may reflect not only inequities in research infrastructure but also the relative scarcity of standardized, manualized community mental health interventions available for evaluation in these contexts. Where such interventions are still being established, the priorities of process evaluation may differ, centring on feasibility, acceptability, and cultural adaptation rather than fidelity to a predefined model. Researchers in these settings have often drawn on alternative or complementary approaches, including locally grounded participatory and community-based traditions, task-sharing and task-shifting models of the kind advanced through the World Health Organization’s Mental Health Gap Action Programme (mhGAP), and culturally embedded evaluation practices that may not map neatly onto frameworks developed in high-income settings. Recognizing these alternatives, rather than treating implementation science frameworks as a universal default, may better support the development of contextually appropriate process evaluation in global mental health.”

We have added a supporting reference to the WHO mhGAP programme (reference 31) to accompany this addition.

We hope these revisions satisfactorily address the points raised. Thank you again for your consideration.

Sincerely,

Rainer Mere, on behalf of all authors

Attachment Submitted filename: Response_to_Reviewers.docx

# Peer review: Decision Letter 1

**DOI:** 10.1371/journal.pone.0354732.r003

**Authors:** Rogis Baker

12 Jul 2026

From participation to systematization: A scoping review of theoretical frameworks guiding process evaluations in community mental health interventions

PONE-D-26-09912R1

Dear Mere,

We’re pleased to inform you that your manuscript has been judged scientifically suitable for publication and will be formally accepted for publication once it meets all outstanding technical requirements.

Within one week, you’ll receive an e-mail detailing the required amendments. When these have been addressed, you’ll receive a formal acceptance letter and your manuscript will be scheduled for publication.

An invoice will be generated when your article is formally accepted. Please note, if your institution has a publishing partnership with PLOS and your article meets the relevant criteria, all or part of your publication costs will be covered. Please make sure your user information is up-to-date by logging into Editorial Manager at Editorial Manager® and clicking the ‘Update My Information' link at the top of the page. For questions related to billing, please contact billing support.

If your institution or institutions have a press office, please notify them about your upcoming paper to help maximize its impact. If they’ll be preparing press materials, please inform our press team as soon as possible -- no later than 48 hours after receiving the formal acceptance. Your manuscript will remain under strict press embargo until 2 pm Eastern Time on the date of publication. For more information, please contact onepress@plos.org.

Kind regards,

Rogis Baker, Ph.D

Academic Editor

PLOS One

Additional Editor Comments (optional):

Reviewers' comments:

Reviewer's Responses to Questions

**Comments to the Author**

1. If the authors have adequately addressed your comments raised in a previous round of review and you feel that this manuscript is now acceptable for publication, you may indicate that here to bypass the “Comments to the Author” section, enter your conflict of interest statement in the “Confidential to Editor” section, and submit your "Accept" recommendation.

Reviewer #1: All comments have been addressed

Reviewer #2: All comments have been addressed

**********

2. Is the manuscript technically sound, and do the data support the conclusions?

The manuscript must describe a technically sound piece of scientific research with data that supports the conclusions. Experiments must have been conducted rigorously, with appropriate controls, replication, and sample sizes. The conclusions must be drawn appropriately based on the data presented.

Reviewer #1: Yes

Reviewer #2: Yes

**********

3. Has the statistical analysis been performed appropriately and rigorously?

Reviewer #1: Yes

Reviewer #2: Yes

**********

4. Have the authors made all data underlying the findings in their manuscript fully available?

The PLOS Data policy requires authors to make all data underlying the findings described in their manuscript fully available without restriction, with rare exception (please refer to the Data Availability Statement in the manuscript PDF file). The data should be provided as part of the manuscript or its supporting information, or deposited to a public repository. For example, in addition to summary statistics, the data points behind means, medians and variance measures should be available. If there are restrictions on publicly sharing data—e.g. participant privacy or use of data from a third party—those must be specified.

Reviewer #1: Yes

Reviewer #2: Yes

**********

5. Is the manuscript presented in an intelligible fashion and written in standard English?

PLOS ONE does not copyedit accepted manuscripts, so the language in submitted articles must be clear, correct, and unambiguous. Any typographical or grammatical errors should be corrected at revision, so please note any specific errors here.

Reviewer #1: Yes

Reviewer #2: Yes

**********

6. Review Comments to the Author

Please use the space provided to explain your answers to the questions above. You may also include additional comments for the author, including concerns about dual publication, research ethics, or publication ethics. (Please upload your review as an attachment if it exceeds 20,000 characters)

Reviewer #1: I have no further comments or revisons , I recommend this muscript for publication in PLOS journal.

Reviewer #2: Both comments raised have been adequately addressed and incorporated in the manuscript along with appropriate reference.

**********

7. PLOS authors have the option to publish the peer review history of their article (what does this mean?). If published, this will include your full peer review and any attached files.

If you choose “no”, your identity will remain anonymous but your review may still be made public.

**Do you want your identity to be public for this peer review?**  For information about this choice, including consent withdrawal, please see our Privacy Policy.

Reviewer #1: No

Reviewer #2: **Yes:**  Amit Chakrabarti

**********

# Editor report: Acceptance letter

**DOI:** 10.1371/journal.pone.0354732.r004

**Authors:** Rogis Baker

PONE-D-26-09912R1

PLOS One

Dear Dr. Mere,

I'm pleased to inform you that your manuscript has been deemed suitable for publication in PLOS One. Congratulations! Your manuscript is now being handed over to our production team.

At this stage, our production department will prepare your paper for publication. This includes ensuring the following:

* All references, tables, and figures are properly cited

* All relevant supporting information is included in the manuscript submission,

* There are no issues that prevent the paper from being properly typeset

You will receive further instructions from the production team, including instructions on how to review your proof when it is ready. Please keep in mind that we are working through a large volume of accepted articles, so please give us a few days to review your paper and let you know the next and final steps.

Lastly, if your institution or institutions have a press office, please let them know about your upcoming paper now to help maximize its impact. If they'll be preparing press materials, please inform our press team within the next 48 hours. Your manuscript will remain under strict press embargo until 2 pm Eastern Time on the date of publication. For more information, please contact onepress@plos.org.

You will receive an invoice from PLOS for your publication fee after your manuscript has reached the completed accept phase. If you receive an email requesting payment before acceptance or for any other service, this may be a phishing scheme. Learn how to identify phishing emails and protect your accounts at https://explore.plos.org/phishing.

If we can help with anything else, please email us at customercare@plos.org.

Thank you for submitting your work to PLOS ONE and supporting open access.

Kind regards,

PLOS ONE Editorial Office Staff

on behalf of

Dr. Rogis Baker

Academic Editor

PLOS One
